# Chitooligosaccharide/Polydopamine Co-Deposition Modifying Substrates for High-Performance Forward Osmosis Membranes with Enhanced Antibacterial and Antifouling Properties

**DOI:** 10.3390/membranes16060186

**Published:** 2026-05-28

**Authors:** Ming-Xiao Zhang, Rui Han, Zhen-Liang Xu, Xin Zhang, Dibakar Pandaya

**Affiliations:** 1 State Key Laboratory of Chemical Engineering, Membrane Science and Engineering R&D Lab, Chemical Engineering Research Center, School of Chemical Engineering, East China University of Science and Technology, 130 Meilong Road, Shanghai 200237, China; 2 Shanghai Electronic Chemicals Innovation Institute, East China University of Science and Technology, Shanghai 200237, China; 3 Beijing Key Laboratory for Membrane Materials and Engineering, Department of Chemical Engineering, Tsinghua University, Beijing 100084, China

**Keywords:** forward osmosis, chitooligosaccharide, polydopamine, co-deposition, polyamide, antibacterial, antifouling

## Abstract

Forward osmosis (FO) membranes have garnered widespread research interest in water treatment, yet their permeability–selectivity trade-off, internal concentration polarization, and membrane fouling remain critical challenges. Herein, a chitooligosaccharide/polydopamine (COS/PDA) co-deposition strategy was proposed to modify polyethersulfone (PES) substrates for constructing high-performance thin-film composite (TFC) FO membranes. COS suppressed excessive PDA aggregation, reduced substrate roughness, and improved substrate hydrophilicity. This substrate modification regulated interfacial polymerization by increasing the adsorption capacity for m-phenylenediamine (MPD) while slowing its diffusion rate, thereby forming thinner, smoother, and more densely crosslinked polyamide (PA) layers. The optimized C_4_P_1_-TFC membrane delivered water fluxes of 42.2 and 23.5 L m^−2^ h^−1^ in pressure-retarded osmosis (PRO) and FO modes, respectively, representing 43.1% and 40.2% improvements over the pristine membrane. Its specific salt flux decreased to 0.07 and 0.15 g L^−1^ in the two modes, respectively, suggesting enhanced selectivity. Meanwhile, the C_4_P_1_-TFC membrane showed antibacterial rates of 85.7% against *Escherichia coli* and 86.9% against *Staphylococcus aureus*, together with improved antifouling performance against bovine serum albumin and lysozyme. This work presents a simple and effective co-deposition approach for simultaneously improving the separation, antibacterial, and antifouling performance of TFC FO membranes, showing promising potential for practical applications.

## 1. Introduction

With the accelerating pace of industrialization and urbanization, water pollution and freshwater scarcity have evolved into increasingly serious global issues [1,2]. Membrane separation processes have been widely employed in water treatment due to their high efficiency, modular design, and low operational costs [3,4]. Forward osmosis (FO) technology utilizes an osmotic pressure gradient as the driving force rather than external hydraulic pressure. Unlike traditional pressure-driven membrane technologies, FO requires little or no external hydraulic pressure to induce water transport across the membrane, which can reduce hydraulic energy input [5,6]. In addition, the absence of hydraulic pressure may alleviate foulant compaction on the membrane surface and improve fouling reversibility under certain conditions [6,7]. Therefore, FO has demonstrated broad application prospects in seawater desalination, wastewater treatment, and material concentration [8,9,10,11].

Despite these merits, FO membranes still suffer from drawbacks, including severe internal concentration polarization (ICP), the permeability–selectivity trade-off, and membrane fouling, which significantly hinder their practical applications. Thin-film composite (TFC) membranes have become the dominant membrane architecture for the FO process. They typically consist of a dense polyamide (PA) active layer supported by a porous polymer substrate [12,13], and the above-mentioned drawbacks are closely associated with the synergistic effects of the substrate and PA layer. The substrate not only governs ICP behavior, but also significantly affects the interfacial polymerization (IP) reaction by regulating the adsorption, diffusion and distribution of amine monomers. These effects are critical for forming a thin, dense, and defect-free PA layer [14,15]. The physicochemical properties of the PA layer directly determine water transport, solute selectivity and fouling propensity. Furthermore, substrate properties affect the antifouling behavior in the pressure-retarded osmosis (PRO) mode [16,17,18]. Therefore, considerable efforts have been devoted to altering the polymer composition [19,20,21,22], optimizing fabrication conditions [23,24,25], and conducting surface modification [26,27,28], with the aim of enhancing the structural and functional properties of the substrate and thereby improving the overall performance of TFC FO membranes.

Surface modification is a simple and highly effective technique for tailoring substrate properties. Many materials have been used to modify the substrate surfaces, such as nanomaterials [26,29,30], metal–polyphenol complexes [10,31], and organic macromolecules [1,28,32,33]. Among them, organic macromolecules have emerged as a promising research focus owing to their excellent compatibility with polymer substrates and their ability to achieve multifunctional performance enhancement. Jiang et al. [28] fabricated a poly(vinyl alcohol) (PVA)-glutaraldehyde (GA) hydrogel layer to modify the substrate, which markedly decreased the PA layer thickness and the membrane structural parameter, thus achieving simultaneous optimization of membrane permeability and selectivity. Zhang et al. [1] modified the substrate via a chitosan (CS)/tannic acid (TA) liquid-phase co-deposition strategy. The prepared TFC membrane showed a more uniform surface and enhanced hydrophilicity, thus improving water permeation while suppressing reverse salt transport. Dopamine (DA) can be oxidized at its catechol and amino groups in an alkaline environment, followed by spontaneous self-polymerization to form polydopamine (PDA) [34]. Benefiting from its mussel-inspired universal adhesion, mild deposition conditions and abundant catechol/amine groups, PDA has been demonstrated to improve membrane hydrophilicity and modulate the formation of the PA layer. Arena et al. [35] employed PDA to modify the substrate, thereby enhancing substrate hydrophilicity and promoting wetting behavior. The PDA-TFC membrane exhibited a 4–6-fold increase in water flux relative to its unmodified control. Yang et al. [33] analyzed the role of PDA modification positions in FO performance, and confirmed that surface-deposited PDA coating on the substrate could improve membrane permeability. However, uncontrolled PDA deposition alone may result in excessive aggregate growth, increased membrane surface roughness, pore blockage of the substrate, and the introduction of additional hydraulic resistance [36,37].

Chitooligosaccharide (COS) is a degradation product of CS via chemical or enzymatic methods. It is derived from chitin, which is the second most abundant natural polymer after cellulose. Most commercially available COS is produced by treating shell waste from shrimp, crabs, and other crustaceans, representing a typical green economic process for valorizing waste into value-added products. Bio-derived COS retains the abundant hydroxyl and amino functional groups of chitosan, as well as its good biocompatibility, low toxicity, and intrinsic antibacterial activity. In addition, COS possesses better water solubility and processability than CS, making it suitable for substrate surface modification [38,39,40]. However, COS alone cannot be stably deposited on polymer substrates, limiting its direct application in substrate surface modification. In contrast, PDA can act as an ideal mussel-inspired adhesive platform through its universal adhesion, serving as a bridge to immobilize COS on the substrate surface.

Therefore, a COS/PDA co-deposition strategy was proposed to modify macroporous polyethersulfone (PES) substrates for constructing TFC FO membranes in this study, aiming to expand the application of COS in FO substrate modification. Through Michael addition and/or Schiff base reactions between COS and PDA [41], COS was expected to be stably immobilized on the substrate, while alleviating the pore blockage and additional hydraulic resistance caused by excessive PDA aggregation during conventional sole PDA deposition. More importantly, based on the characteristics of COS, the COS/PDA-tailored substrates were designed not only to optimize the physicochemical properties of the substrate, such as hydrophilicity, surface charge, and pore structure, but also to modulate the mass transfer behavior of m-phenylenediamine (MPD) monomers, thereby affecting the IP process and the formation of the PA active layers. This study aims to clarify how COS/PDA co-deposition alters the surface characteristics of PES substrates and the diffusion behavior of MPD monomers, to reveal how this modification strategy regulates the structure of TFC FO membranes, and to further elucidate its influence on the separation, antifouling, and antibacterial performance of the resultant membranes, providing new insight into the synergistic enhancement of multifunctional membrane performance.

## 2. Materials and Methods

### 2.1. Materials

The PES macroporous substrates (pore size: 0.22 μm) were supplied by Nantong Longjin Membrane Technology Co., Ltd., Nantong, China. COS (molecular weight 1000–2000 Da, degree of deacetylation ≥90%) and lysozyme (LYZ, ≥20,000 units mg^−1^) were provided by Hefei Bomei Biotechnology Co., Ltd., Hefei, China. Dopamine hydrochloride (DA, ≥98%), triethylamine (TEA, ≥99%), tris(hydroxymethyl)aminomethane (Tris, ≥98%), and bovine serum albumin (BSA, ≥96%) were supplied by Shanghai Macklin Biochemical Technology Co., Ltd., Shanghai, China. Trimesoyl chloride (TMC, ≥98%), sodium chloride (NaCl, ≥99.5%), hydrochloric acid (HCl, AR), and n-hexane (≥97%) were acquired from Shanghai Titan Scientific Co., Ltd., Shanghai, China. MPD (≥99%) was obtained from Shanghai Aladdin Biochemical Technology Co., Ltd., Shanghai, China. Phosphate-buffered saline (PBS, 10×) was purchased from Beijing Solarbio Science and Technology Co., Ltd., Beijing, China. *Escherichia coli* (*E. coli*, ATCC 25922) and *Staphylococcus aureus* (*S. aureus*, ATCC 29213) were obtained from the Guangdong Microbial Culture Collection Center, Guangzhou, China. Luria–Bertani (LB) medium was supplied by Sangon Biotech (Shanghai) Co., Ltd., Shanghai, China. Laboratory-prepared deionized (DI) water was utilized in the experiments.

### 2.2. Co-Deposition Modification of PES Substrates

**Figure 1:**
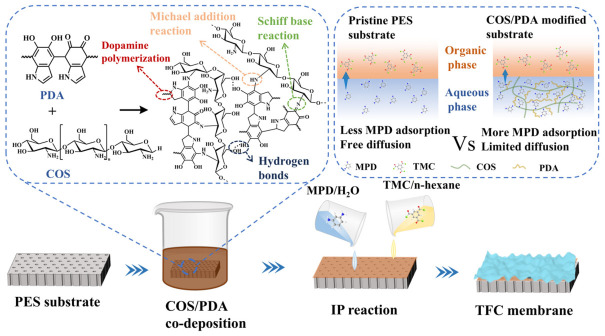
Fabrication schematic diagram of TFC FO membranes with COS/PDA co-deposition modified substrates.

The PES substrates were modified via COS/PDA co-deposition. The preparation process and the reactions involved are presented in Figure 1. To remove glycerol, the substrates were first soaked in DI water for over 12 h. A 50 mM Tris buffer solution at pH 8.5 was prepared using HCl for pH adjustment. The substrates were then immersed in the freshly prepared Tris buffer solution containing DA and COS, and the mixture was shaken at ambient temperature for 4 h. After co-deposition, the obtained substrates were thoroughly washed with DI water, heated at 40 °C for 1 h, and finally stored in DI water at room temperature until use. These modified substrates were denoted as C_x_P_y_-PES, in which x and y refer to the concentrations (g L^−1^) of COS and DA in the mixed solution, respectively. The deposition conditions were optimized, and a DA concentration of 1 g L^−1^ and a co-deposition time of 4 h were chosen for subsequent investigations into the effects of COS concentration (see Section S.1, Figures S1 and S2 in the Supplementary Materials for details).

### 2.3. Fabrication of TFC FO Membranes

The PA active layers were prepared by a conventional IP method, as shown in Figure 1. A substrate was laid on a flat plate, and its edges were secured with a sealing ring. Then, an aqueous solution containing 2 *w*/*v*% MPD and 1 *w*/*v*% TEA was poured onto the substrate surface. Following a contact time of 5 min, the excess aqueous phase was removed, and the substrate surface was rolled using a rubber roller. Subsequently, a 0.15 *w*/*v*% TMC/n-hexane solution was poured onto the MPD-impregnated surface for 1 min to initiate IP. The obtained membrane underwent a 70 °C heat-treatment for 5 min, and was preserved in DI water at ambient temperature before being subjected to subsequent characterization and tests. The resulting TFC FO membranes were denoted as C_x_P_y_-TFC according to their corresponding modified substrates. The TFC FO membrane formed on the unmodified PES substrate was denoted as PES-TFC.

### 2.4. Characterization

The morphology and roughness of substrates and TFC membranes were examined by scanning electron microscopy (SEM, ZEISS GeminiSEM 300, Jena, Germany) and atomic force microscopy (AFM, Bruker Dimension Icon, Karlsruhe, Germany), respectively. The apparent surface pore-opening sizes of the substrates were determined from the SEM images using ImageJ software (version 1.54g). Due to the irregular pore shapes, the pore-opening size was expressed as the equivalent circular diameter calculated from the projected pore-opening area. The chemical functional groups present in the substrates and membranes were analyzed via an attenuated total reflection Fourier transform infrared spectrometer (ATR-FTIR, Thermo Fisher Scientific Nicolet iS20, Waltham, MA, USA). The atomic compositions were characterized by X-ray photoelectron spectroscopy (XPS, Thermo Scientific K-Alpha, Waltham, MA, USA). A contact angle test system (JC2000A, Zhong Chen, Shanghai, China) was utilized to evaluate the hydrophilicity of substrates and membranes. The zeta potentials of the substrates and membranes were investigated by an electrokinetic analyzer (SurPASS 3, Anton Paar, Graz, Austria). The absorbance of MPD diffused into n-hexane was recorded with ultraviolet–visible spectroscopy (UV-vis, Shimadzu UV-2700, Kyoto, Japan).

### 2.5. Diffusion of MPD

To clarify the role of COS in regulating the IP process, MD simulations were employed to compare the diffusion behavior of MPD monomers in aqueous systems with and without COS. The simulations were carried out with the open-source software GROMACS 2020.6 [42]. The simulation results were visualized using VMD software (version 1.9.3) [43]. The diffusion coefficient (*D*) of MPD was calculated from the mean squared displacement (MSD) [44]. Furthermore, UV-vis spectroscopy was employed to quantify the absorbance variations in MPD diffusing into n-hexane over time, thereby analyzing the relative diffusion rate of MPD [45,46]. Detailed simulation and experimental methods are provided in Sections S.2 and S.3 in the Supplementary Materials.

### 2.6. Pure Water Permeance of Substrates and Separation Performance of Membranes

A lab-made cross-flow filtration device was used to determine the pure water permeance (PWP, L m^−2^ h^−1^ bar^−1^, LMH bar^−1^) of the substrates. Test details and corresponding calculation equation are provided in Section S.4 in the Supplementary Materials.

The testing conditions and calculation equations for FO performance including water flux (*J*_w_, L m^−2^ h^−1^, LMH) and reverse salt flux (*J*_s_, g m^−2^ h^−1^, gMH), and for intrinsic separation properties including the pure water permeability coefficient (*A*, LMH bar^−1^), the salt permeability coefficient (*B*, LMH), the salt rejection (*R*, %), and the structural parameter (*S*, μm), are detailed in Sections S.5 and S.6 in the Supplementary Materials.

### 2.7. Antibacterial and Antifouling Performance of Membranes

*E. coli* and *S. aureus* were chosen as representative bacterial strains to assess the antibacterial performance of the membranes via the spread plate counting method. In addition, the antifouling performance of the membranes was investigated by dynamic fouling tests in both FO and PRO modes, using 200 mg L^−1^ BSA (negatively charged) and 200 mg L^−1^ LYZ (positively charged) as model foulants. During the dynamic fouling tests, the foulant-containing solution was used as the feed solution, while 1 mol L^−1^ NaCl served as the draw solution. The water flux was continuously recorded to evaluate the flux decline of the membranes. After the fouling operation, the fouled membranes were rinsed with DI water for 30 min, and the water flux after cleaning was measured to evaluate the flux recovery behavior. The specific procedures and calculation equations for the antibacterial rate (AR, %) [47,48], flux decline ratio (FDR, %), and flux recovery ratio (FRR, %) are detailed in Sections S.7 and S.8 in the Supplementary Materials.

## 3. Results and Discussion

### 3.1. Structures and Properties of Substrates

**Figure 2:**
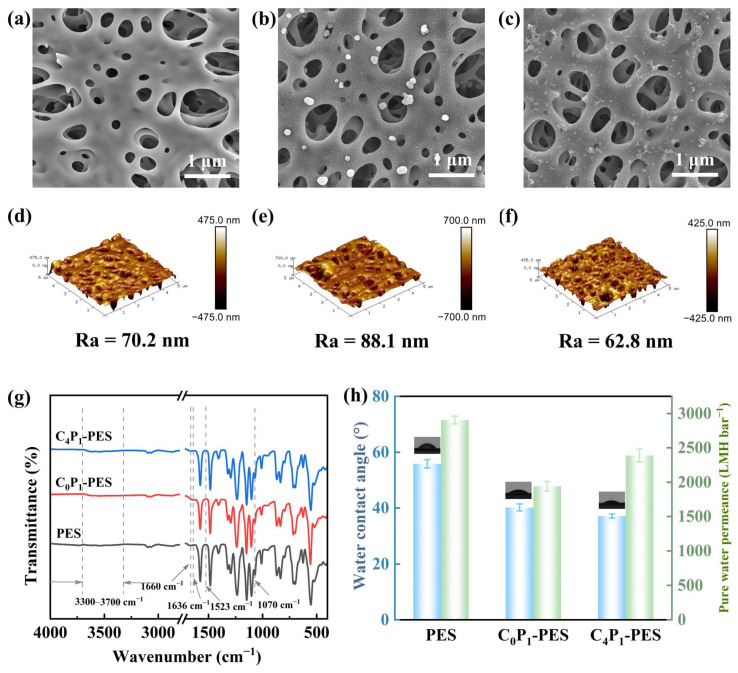
Surface SEM images and AFM images of (**a**,**d**) pristine PES substrate, (**b**,**e**) C_0_P_1_-PES substrate, and (**c**,**f**) C_4_P_1_-PES substrate; (**g**) FTIR spectra, and (**h**) WCAs and PWP of different substrates, with the insets showing representative water droplet profiles used for WCA measurements.

As presented in Figure 2a–f, the surface morphologies of substrates underwent significant changes following COS/PDA co-deposition. The pristine commercial PES substrate was porous and exhibited distinct pits and undulations. The average apparent pore-opening size of the pristine PES substrate was 0.24 ± 0.08 μm, which was close to the nominal pore size of 0.22 μm provided by the supplier. In a weakly alkaline environment, DA formed aggregates that grew continuously with increasing reaction time via oxidative self-polymerization. When DA was deposited alone on the substrate surface, some spherical PDA nanoclusters were observed (see Figure 2b), leading to increased surface roughness. The Ra value increased from 70.2 nm for the pristine PES substrate to 88.1 nm for the C_0_P_1_-PES substrate. Some of these nanoclusters partially entered the microporous channels on the substrate surface, reducing the average apparent pore-opening size to 0.19 ± 0.05 μm. When COS and DA were co-deposited, COS was introduced onto the substrate surface through covalent reactions with PDA, as shown in Figure 1. This reaction regulated the assembly process of PDA, disrupted the non-covalent interactions among PDA aggregates, and inhibited excessive PDA aggregation [49]. Consequently, a smooth and uniform COS/PDA coating without large protrusions was formed on the substrate surface. This resulted in a reduced Ra value of 62.8 nm and helped alleviate the blockage of surface micropores, with the C_4_P_1_-PES substrate exhibiting an average apparent pore-opening size of 0.22 ± 0.04 μm.

The chemical compositions of the substrate surface were characterized by FTIR spectroscopy. As illustrated in Figure 2g, compared with the pristine PES substrate, the C_0_P_1_-PES substrate exhibited a broad characteristic absorption peak at approximately 3300–3700 cm^−1^, attributed to the O–H and N–H stretching vibrations. This confirmed the successful introduction of hydrophilic groups onto the substrate surface. This peak was more pronounced on the C_4_P_1_-PES substrate, which was attributed to the superposition of numerous hydroxyl groups in COS and the enhanced intermolecular hydrogen bonding. The absorption peak at 1636 cm^−1^ for the C_0_P_1_-PES substrate was assigned to the stretching vibration of quinone C=O groups generated by PDA oxidation. After COS addition, the intensity of this peak decreased, and a C=N stretching vibration peak appeared at 1660 cm^−1^, suggesting that a Schiff base reaction occurred between COS and PDA. Furthermore, the increased intensity of the N–H bending vibration peak at 1523 cm^−1^ for the C_4_P_1_-PES substrate could be associated with the formation of more secondary amines via a Michael addition reaction between COS and PDA. The peak located at 1030 cm^−1^ corresponded to the stretching vibration of C–O–C bonds in the pyranose rings of COS. All these changes indicated that COS and PDA were successfully co-deposited on the PES substrate surface.

As shown in Figure 2h, co-deposition modification clearly improved the hydrophilicity of the substrate surface. The water contact angle (WCA) decreased from 55.5° for the pristine PES substrate to 40.0° (C_0_P_1_-PES) and 37.3° (C_4_P_1_-PES), respectively. In particular, the COS/PDA co-deposition introduced a high density of hydrophilic functional groups, which could strongly interact with water molecules through hydrogen bonding, thus accelerating the formation of a hydration layer and significantly improving the hydrophilicity of the C_4_P_1_-PES substrate [50]. The WCAs of C_0_P_1_-PES and C_4_P_1_-PES did not differ significantly, which was mainly because the higher surface roughness of C_0_P_1_-PES amplified its apparent hydrophilicity through the Wenzel effect [51]. Generally, better surface hydrophilicity strengthens the affinity between the substrate and water, which promotes the wetting, spreading and permeation of water molecules [52]. However, for porous substrates, PWP is not governed solely by surface hydrophilicity, but is also affected by the pore size and pore blockage. When PDA was deposited alone, although the surface hydrophilicity of the substrate was enhanced, SEM images showed that some PDA nanoclusters entered the surface micropores, resulting in a reduced average apparent pore-opening size. The blockage of surface micropores increased the water permeation resistance and thus lowered the PWP value. In contrast, the COS/PDA co-deposition improved the surface hydrophilicity while effectively suppressing excessive formation of PDA aggregates and alleviating pore blockage, thereby mitigating the reduction in PWP. These findings indicated that COS/PDA co-deposition modification significantly modulated the surface structure and physicochemical properties of the substrates, which would affect the subsequent IP process and endow the TFC membranes with distinct separation and antifouling performance.

### 3.2. Chemical Structures and Hydrophilicity of TFC FO Membranes

**Figure 3:**
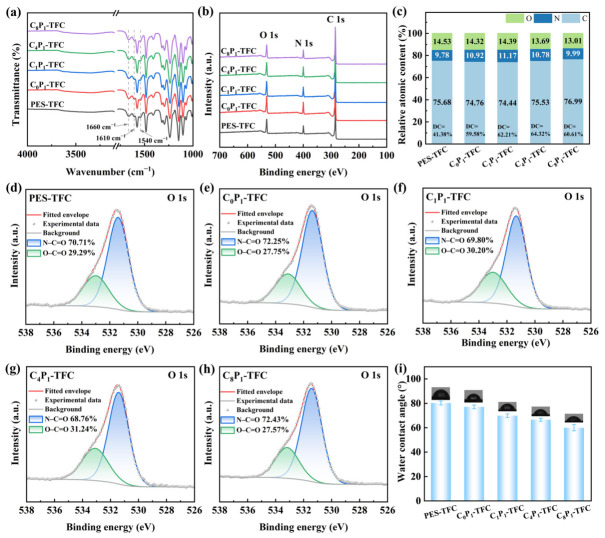
(**a**) FTIR spectra, (**b**) wide-scan XPS spectra, and (**c**) atomic content and crosslinking degree of the TFC membranes; high-resolution O 1s spectra for (**d**) PES-TFC, (**e**) C_0_P_1_-TFC, (**f**) C_1_P_1_-TFC, (**g**) C_4_P_1_-TFC, and (**h**) C_8_P_1_-TFC; (**i**) WCAs of the TFC membranes, with the insets showing representative water droplet profiles used for WCA measurements.

The chemical structures of C_x_P_1_-TFC FO membranes fabricated on different COS/PDA modified substrates were characterized by FTIR spectroscopy. As presented in Figure 3a, all TFC membranes displayed three distinct characteristic absorption peaks at 1660 cm^−1^, 1610 cm^−1^ and 1540 cm^−1^. Among these, the peak at 1660 cm^−1^ was assigned to the C=O stretching vibration of the amide I band in the PA layer. The peak at 1540 cm^−1^ corresponded to the N–H bending vibration and C–N stretching vibration of the amide II band, while the peak at 1610 cm^−1^ originated from the C=C stretching vibration of aromatic rings. Although the COS/PDA co-deposition on the substrate surface already exhibited characteristic absorption peaks in relevant regions (such as Schiff base or secondary amine vibrations), the significantly increased intensity of these absorption peaks, together with the characteristic features of the PA fingerprint region, collectively confirmed that the PA layers were successfully formed on the modified substrates.

XPS characterization was performed to further investigate the surface elemental and chemical compositions of the PA layers in different TFC FO membranes. Figure 3b presents the wide-scan XPS spectra of the prepared C_x_P_1_-TFC FO membranes. For all membrane samples, three peaks were observed at approximately 285 eV, 400 eV, and 531 eV, corresponding to C 1s, N 1s, and O 1s orbitals, respectively. As described in Section S.9 in the Supplementary Materials, the degree of crosslinking (DC) was evaluated based on the O/N ratio [53], and the calculated results are given in Figure 3c. The DC value of the PES-TFC membrane was 41.18%. With increasing COS concentration, the crosslinking degree exhibited an initial increase followed by a decrease, while the C_1_P_1_-TFC and C_4_P_1_-TFC membranes demonstrated relatively higher crosslinking degrees. This trend could be attributed to the regulation of the IP process by the COS/PDA modified substrates. COS/PDA co-deposition enhanced the hydrophilicity of the substrates, and a higher COS concentration led to a higher density of hydrophilic groups, thus enabling the adsorption of more MPD monomers to participate in the IP reaction and ultimately forming a more densely crosslinked PA layer. This interpretation was consistent with previous reports that substrate hydrophilicity and amine monomer adsorption could regulate PA layer formation during IP [54]. Meanwhile, hydrogen bonds between COS and MPD limited the diffusion rate of MPD. These two effects are illustrated in Figure 1. However, at excessively high COS concentrations, the O/N ratio increased. This was likely related to the excessive inhibition of MPD diffusion by excess COS, which led to an insufficient supply or uneven release of MPD at the reaction interface. Furthermore, excess COS components may remain near the interface, or their un-crosslinked active amino groups may undergo local reactions with TMC, contributing to the increase in the O/N ratio. High-resolution O 1s spectra are shown in Figure 3d–h. For the C_0_P_1_-TFC membrane, the proportion of N–C=O was higher than that in the PES-TFC membrane, which was in accordance with its higher crosslinking degree. In contrast, the proportion of O–C=O in the C_1_P_1_-TFC and C_4_P_1_-TFC membranes increased. This could be explained by the enrichment of MPD at the interface of the modified substrates, which promoted PA network formation. Compared with the sole PDA deposition system, COS significantly slowed down the diffusion of MPD monomers, as discussed in detail in Section 3.4. This diffusion regulation may result in an increased number of unreacted acyl chloride groups at the chain termini of the growing PA network. While the majority of these terminal acyl chlorides continued to react with MPD to extend the crosslinked network, a small fraction of unreacted groups hydrolyzed to form carboxyl groups. The reduced O–C=O proportion in the C_8_P_1_-TFC membrane was possibly ascribed to the partial residual COS near the interface or the consumption of acyl chloride groups by partial COS participating in the IP process, as analyzed above.

WCA tests were carried out on the surfaces of the TFC FO membranes to characterize and compare their surface hydrophilicity, as presented in Figure 3i. With increasing COS concentration, the WCAs showed an overall downward trend. The WCA of the PES-TFC membrane was 80.2°, while the value for C_4_P_1_-TFC dropped to 66.5°. This was consistent with its increased O–C=O proportion revealed by XPS characterization results. C_8_P_1_-TFC displayed the lowest WCA of 59.5°, which indirectly suggested that a number of hydroxyl and amino groups from COS may have been incorporated into the PA layer through chemical bonding and physical entanglement. The optimization of membrane hydrophilicity could strengthen the interaction between the membrane surface and water molecules, thereby reducing foulant adsorption and adhesion and contributing to enhanced membrane antifouling performance.

### 3.3. Morphologies of TFC FO Membranes

**Figure 4:**
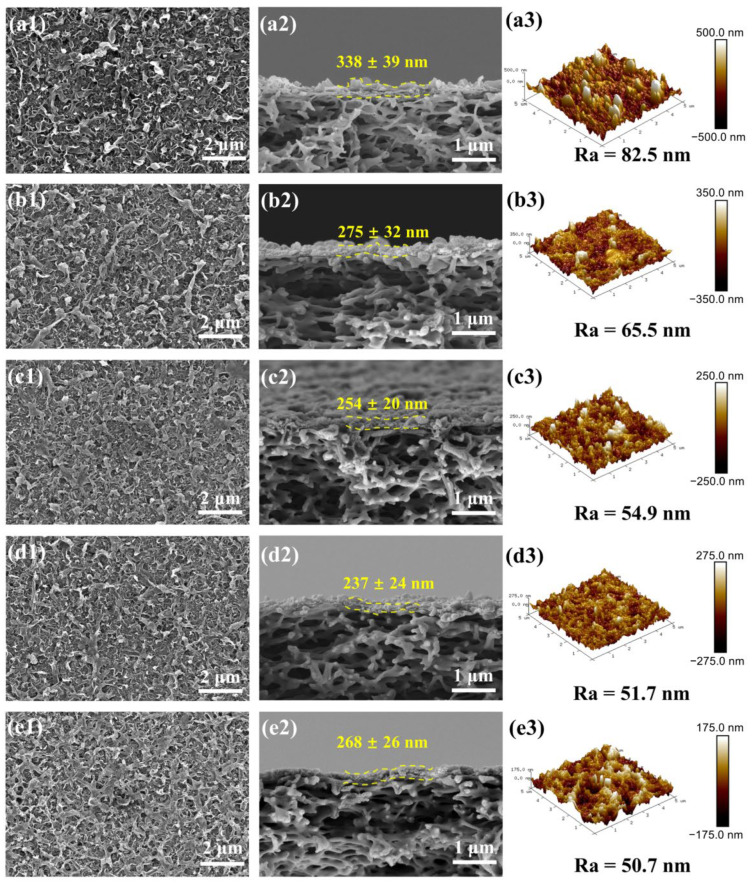
Surface, cross-sectional SEM morphologies, and AFM images of (**a1**–**a3**) PES-TFC, (**b1**–**b3**) C_0_P_1_-TFC, (**c1**–**c3**) C_1_P_1_-TFC, (**d1**–**d3**) C_4_P_1_-TFC, and (**e1**–**e3**) C_8_P_1_-TFC membranes.

The surface and cross-sectional morphologies of PES-TFC and C_x_P_1_-TFC FO membranes were observed using SEM. Meanwhile, the surface roughness was measured using AFM. According to Figure 4, the SEM results indicated that the PA layers of all membranes completely covered the microporous structures of the substrates, forming continuous and intact active layers. A typical rough “ridge-and-valley” structure was observed on the PES-TFC membrane, while the surfaces of the C_x_P_1_-TFC series membranes displayed more leaf-like structures. As the concentration of COS used for co-deposition increased, the leaf-like structures became more extended, and the membrane surfaces became more uniform and regular. In the case of the C_8_P_1_-TFC membrane, as mentioned in the XPS analysis, excessive inhibition of MPD diffusion by COS, combined with the possible participation of COS-related active groups in the IP reaction, may have reduced the crosslinking degree of the PA layer. As a result, the originally dense membrane surface exhibited some localized pores or relatively loose regions.

As more COS participated in the co-deposition, the PA layers became gradually thinner (see Figure 4(a2–e2)). This was because MPD monomers formed hydrogen bonds and other intermolecular interactions with PDA, COS, as well as their crosslinked products, which restricted MPD diffusion toward the reaction interface and promoted the formation of a thinner PA layer. Furthermore, the improved substrate hydrophilicity increased the MPD adsorption capacity of the substrates. The higher initial MPD concentration facilitated the formation of a dense initial PA layer, which impeded further MPD diffusion and slowed subsequent PA layer growth [55,56]. However, when the COS concentration was too high, the thickness of the PA layer slightly increased again. This could be attributed to the reduced density and continuity of the initially formed PA layer, which weakened its self-limiting effect. Nevertheless, the PA layer of the C_8_P_1_-TFC membrane remained thinner than that of the PES-TFC membrane.

The AFM results (see Figure 4(a3–e3)) showed that the surface roughness of membranes decreased with increasing COS concentration involved in co-deposition. On the one hand, COS/PDA co-deposition effectively reduced the intrinsic roughness of the substrate, and the “imprinting effect” contributed to a smoother surface of PA layer [57,58]. On the other hand, the restricted diffusion of MPD resulted in more uniform MPD release, thereby preventing surface protrusions caused by excessively fast local polymerization rates and facilitating the generation of a smooth and uniform PA layer.

### 3.4. Diffusion Behavior of MPD

**Figure 5:**
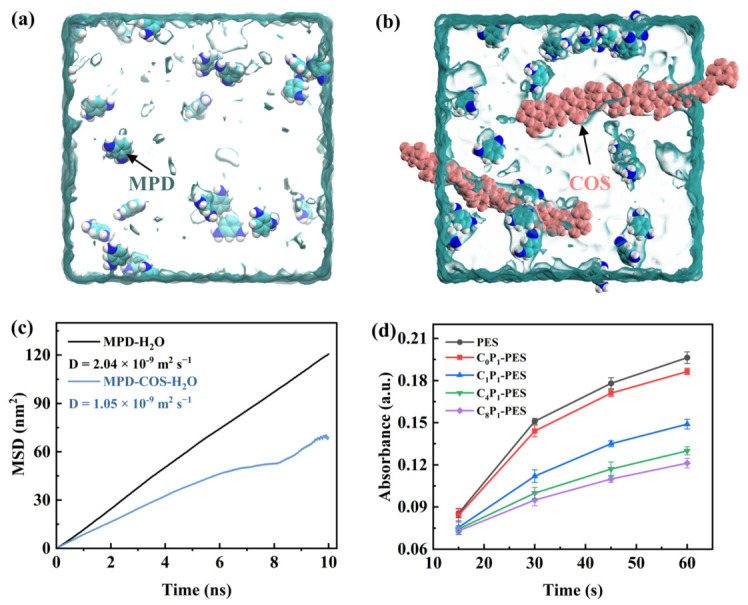
MD simulation models of (**a**) MPD-H_2_O system, (**b**) MPD-COS-H_2_O system; (**c**) MSD curves and *D* of MPD in two systems; and (**d**) Absorbance of MPD diffused from different substrates into n-hexane.

The influence of the substrate on amine monomer diffusion is critical for regulating the physicochemical properties and morphology of the PA layer [59,60]. MD simulations were carried out to examine the effect of COS on the diffusion rate of MPD, as presented in Figure 5a,b. Figure 5c displays the diffusion coefficients of MPD derived from the MSD curves. The calculated results indicated that the presence of COS reduced the diffusion coefficient of MPD from 2.04 × 10^−9^ m^2^ s^−1^ to 1.05 × 10^−9^ m^2^ s^−1^. The main reasons for this phenomenon were as follows: the abundant hydroxyl and amino groups in COS molecules formed hydrogen bonds with MPD molecules, restricting the mobility of MPD molecules. Meanwhile, the COS molecular chains increased steric hindrance within the system, limiting the diffusive mobility of MPD molecules and thus resulting in a reduced diffusion rate.

To experimentally evaluate the regulatory effect of COS/PDA co-deposited substrates at different COS concentrations on the MPD diffusion rate, UV-vis spectroscopy was employed to monitor the absorbance variations in MPD diffusing from different substrates into n-hexane over time. By comparing the slopes of the absorbance-time curves in Figure 5d, the presence of COS was found to further inhibit the MPD diffusion. Further analysis of the trends at different COS concentrations demonstrated that, when the COS concentration increased to 1 g L^−1^, the absorbance decreased significantly, indicating that even a small amount of COS was sufficient to alter the mass transfer characteristics of the substrate surface. With a further rise in COS concentration to 4 g L^−1^, the diffusion of MPD was more strongly restricted, and the absorbance continued to decrease. However, when the COS concentration reached 8 g L^−1^, the difference caused by further increasing the COS concentration became less pronounced. Since the crosslinking reaction between COS and PDA may have tended to stabilize at a fixed PDA concentration, the further reduction in the apparent diffusion rate of MPD caused by increasing COS concentration was relatively limited. In conclusion, COS/PDA co-deposition of the substrates could effectively regulate the diffusion process of MPD monomers and consequently affect the morphology and separation performance of the resultant membranes.

### 3.5. Separation Performance of TFC FO Membranes

**Figure 6:**
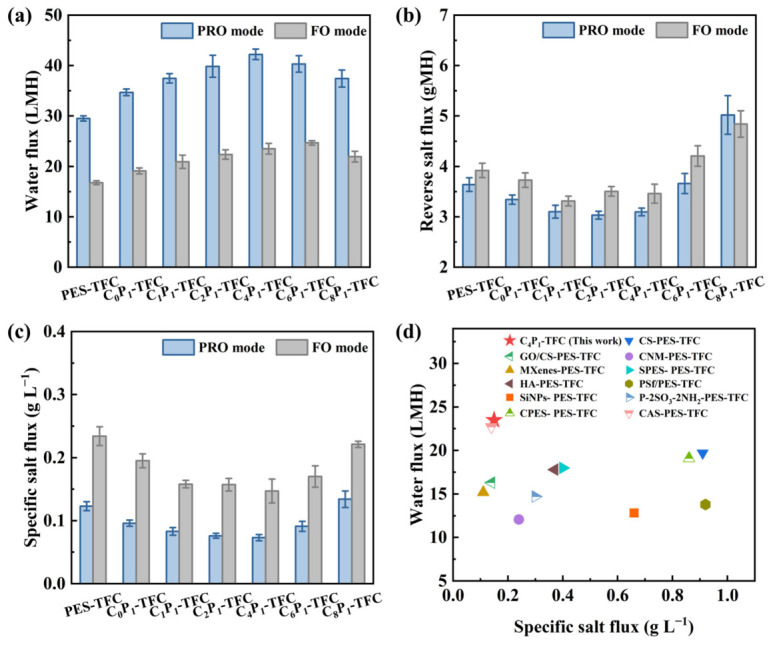
FO performance of PES-TFC and C_x_P_1_-TFC series membranes: (**a**) water flux, (**b**) reverse salt flux, and (**c**) specific salt flux; (**d**) FO performance comparison between C_4_P_1_-TFC and other reported PES-based TFC membranes.

The FO performance of the fabricated membranes was measured and compared in PRO and FO modes. As shown in Figure 6a, the water flux of the PES-TFC membrane was relatively low. After PDA deposition, the C_0_P_1_-TFC membrane exhibited improved water flux. With increasing COS concentration in the co-deposition modification, the water flux continued to increase. The C_4_P_1_-TFC membrane exhibited the highest water fluxes, reaching 42.2 and 23.5 LMH in PRO and FO modes, respectively. This improvement was mainly attributed to the synergistic effects of two factors. First, COS/PDA co-deposition modification enhanced substrate hydrophilicity without compromising the original porous structure, which helped alleviate the ICP effect [61]. Second, as indicated by the characterization results, the modified substrates enabled the fabrication of thinner, smoother, and more hydrophilic PA layers, reducing the resistance to water transport and further promoting water permeation [62]. When the COS concentration was further increased, the water flux ceased to rise, and the C_8_P_1_-TFC membrane showed a significant decrease. This indicated that a higher COS concentration was not always beneficial, as excessive COS failed to optimize the IP process. Instead, it led to a thicker PA layer, raising the resistance to water transport. It simultaneously triggered an increase in reverse salt flux, which lowered the effective osmotic driving force across the membrane. Moreover, the water fluxes in the PRO and FO modes exhibited similar trends. However, the enhancement effect of water flux in the FO mode was less pronounced, which was mainly attributed to its more severe ICP effect [63].

Figure 6b shows the reverse salt flux of the membranes. The PES-TFC membrane and the TFC membranes prepared via co-deposition modification with low COS concentrations exhibited generally low reverse salt fluxes in both modes. When the COS concentration increased, two competing effects existed simultaneously. On the positive side, more MPD was adsorbed on the substrate and participated in the IP reaction, promoting the formation of a dense PA layer. On the negative side, excessive COS competed with MPD for TMC monomers, leading to a decrease in the crosslinking degree of the PA layer. As the negative effect gradually became dominant, the reverse salt flux increased. Specific salt flux (*J*_s_/*J*_w_) is widely adopted as an indicator for evaluating the separation efficiency of FO membranes. A smaller *J*_s_/*J*_w_ value indicates higher membrane selectivity, less solute loss, and lower cost of draw solution replenishment [64]. Figure 6c shows that the C_4_P_1_-TFC membrane achieved the lowest specific salt flux. In comparison with the PES-TFC membrane, its specific salt fluxes decreased from 0.12 and 0.23 g L^−1^ to 0.07 and 0.15 g L^−1^ in PRO and FO modes, respectively, indicating that the C_4_P_1_-TFC membrane possessed superior permeability and selectivity.

Table 1 summarizes the intrinsic separation properties of the prepared membranes and other representative PES-based TFC FO membranes reported in the literature. Compared with the pristine PES-TFC membrane, the C_4_P_1_-TFC membrane exhibited an increased pure water permeability coefficient (*A*) from 1.65 to 2.05 LMH bar^−1^, which was attributed to the improved substrate hydrophilicity and the thinner PA layer. As demonstrated in previous studies [65,66], a higher *A* value is beneficial for enhancing water flux in the FO process, while a lower salt permeability coefficient (*B*) helps suppress reverse salt diffusion and maintain the effective osmotic driving force. Owing to the more highly crosslinked PA layer of the C_4_P_1_-TFC membrane, its *B* value slightly decreased, and the salt rejection (*R*) increased. These results were consistent with the improved water flux and low reverse salt flux of the C_4_P_1_-TFC membrane observed in both PRO and FO modes. A lower structural parameter (*S*) was observed for the C_4_P_1_-TFC membrane, which was related to the higher “effective porosity” of the substrate resulting from the enhanced hydrophilicity after COS/PDA co-deposition modification [67]. In addition, Zhou et al. [68] demonstrated that the structural parameter of an FO membrane is not solely determined by the intrinsic properties of the substrate; the morphology and internal structure of the PA layer can also affect the structural parameter. Therefore, the thinner and smoother PA layer of the C_4_P_1_-TFC membrane could also contribute to its lower structural parameter, which indicated a reduced degree of ICP and improved separation performance.

Compared with other reported PES-based TFC membranes, the C_4_P_1_-TFC membrane achieved a favorable combination of relatively high water permeability, low salt permeability, and high salt rejection. Although some membranes exhibited higher A values, they also showed higher salt permeability. In terms of the structural parameter, the S value of the C_4_P_1_-TFC membrane was 355 μm, which was relatively low among the compared membranes. This comparison further demonstrated that COS/PDA co-deposition was an effective strategy for improving the intrinsic separation properties and mitigating ICP in PES-based TFC FO membranes.

The FO performance of the C_4_P_1_-TFC membrane was compared with other reported FO membranes prepared using PES substrates, and the detailed comparison results are presented in Figure 6d and Table 2. All compared performance data were obtained in the FO mode using DI water as the feed solution and 1 mol L^−1^ NaCl solution as the draw solution. The comparison revealed that the C_4_P_1_-TFC membrane achieved a higher water flux while ensuring low reverse salt flux and specific salt flux. This result highlighted the effectiveness of the COS/PDA co-deposition modification strategy employed in this work.

### 3.6. Antibacterial Performance of TFC FO Membranes

**Figure 7:**
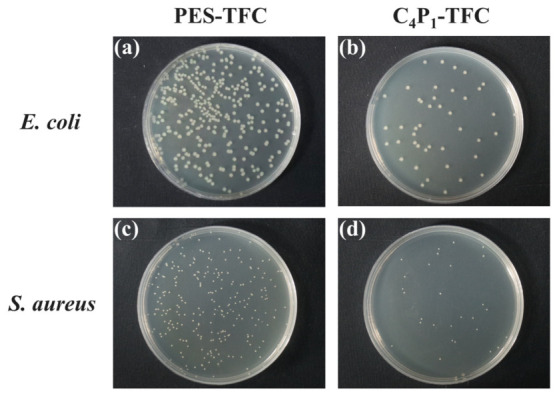
Comparison of antibacterial activity of PES-TFC membrane (**a**,**c**) and C_4_P_1_-TFC membrane (**b**,**d**) by the spread plate counting method.

COS exhibits significant antibacterial activity. It can form an adsorbed layer on the surfaces of bacteria, hindering nutrient transport and normal cellular metabolism. Low-molecular-weight COS can penetrate the bacterial cells, further inhibiting gene transcription and protein synthesis. Meanwhile, the amino groups of COS can readily become protonated and positively charged, enabling electrostatic interactions with negatively charged components on the bacterial cell walls. This disrupts the cell membrane structure and inhibits bacterial growth and reproduction [39]. As shown in Figure 7, compared with the PES-TFC membrane, the C_4_P_1_-TFC membrane achieved antibacterial rates of approximately 85.7% against *E. coli* and 86.9% against *S. aureus*. It should be noted that, because COS/PDA co-deposition was performed on the PES substrate before IP, the antibacterial property was mainly introduced on the support layer side rather than on the PA layer. Therefore, the observed antibacterial performance should be attributed primarily to the exposed COS/PDA-modified substrate. Further surface engineering of the PA layer could provide an additional strategy for achieving dual-sided antibacterial protection.

### 3.7. Antifouling Performance of TFC FO Membranes

**Figure 8:**
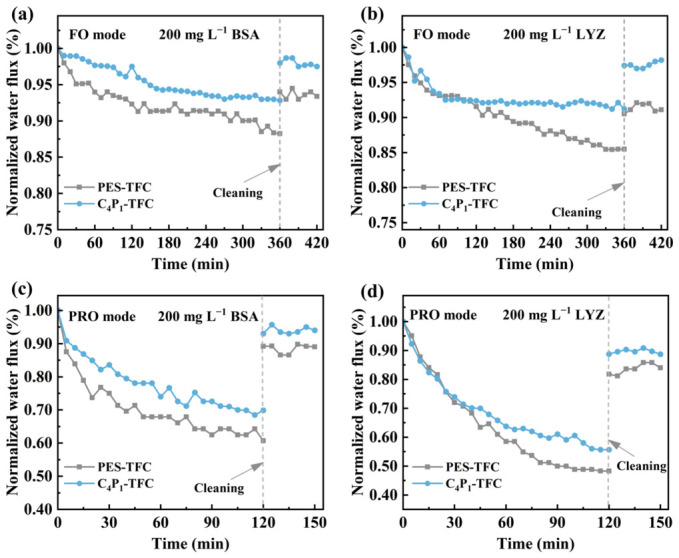
Dynamic fouling tests of PES-TFC membrane and C_4_P_1_-TFC membrane with (**a**) 200 mg L^−1^ BSA in FO mode; (**b**) 200 mg L^−1^ LYZ in FO mode; (**c**) 200 mg L^−1^ BSA in PRO mode; and (**d**) 200 mg L^−1^ LYZ in PRO mode.

The antifouling performance of FO membranes is of great significance, as it is closely associated with their operational stability, service life, and treatment efficiency in practical applications. In this study, BSA and LYZ were employed as typical model foulants, and dynamic fouling tests were conducted in both FO and PRO modes. The test results are presented in Figure 8. In the FO mode, the C_4_P_1_-TFC membrane exhibited a slower decline in water flux and a higher flux recovery ratio against both BSA and LYZ fouling. After 360 min of dynamic fouling testing, when BSA solution was utilized as the feed solution, the flux decline ratios of the PES-TFC and C_4_P_1_-TFC membranes were 11.3% and 7.2%, respectively. After simple physical cleaning of the fouled membranes with DI water for 30 min, their flux recovery ratios were 93.3% and 98.0%, respectively. When LYZ solution was applied as the feed solution, the water fluxes of the two membranes decreased by 14.5% and 8.8%, respectively, and after cleaning, the water fluxes recovered to 90.5% and 97.1% of their initial values, respectively. In FO mode, the feed solution was directly exposed to the PA active layer, and membrane fouling behavior was primarily regulated by the surface properties of the PA layer. The superior hydrophilicity of the PA layer in the C_4_P_1_-TFC membrane helped form a stable hydration layer on the membrane surface, thereby effectively weakening the interfacial interactions between foulants and the membrane surface [82,83]. The smoother surface reduced the number of microscopic retention sites for foulants, significantly weakening their adsorption and deposition on the membrane surface [84]. For BSA fouling, in addition to the aforementioned factors, the enhanced electronegativity of the PA layer surface (see Figure S3) further increased the electrostatic repulsion between negatively charged BSA molecules and the membrane, contributing to the improvement in antifouling performance.

Dynamic fouling tests targeting BSA and LYZ were further conducted for 120 min in the PRO mode. The flux decline for both membranes significantly accelerated, and the decline curves became steeper. This was primarily because the feed solution directly contacted the support layer in the PRO mode, allowing foulants to readily penetrate the porous support layer and thereby aggravating internal fouling [17]. For BSA fouling, the PES-TFC membrane suffered a flux decline ratio of nearly 40%. Owing to the enhanced hydrophilicity and reduced roughness of the support layer (see Figure S3), the flux decline of the C_4_P_1_-TFC membrane was partially mitigated, although it still reached 30.1%. The flux recovery ratio of the C_4_P_1_-TFC membrane reached 92.9%, outperforming that of the PES-TFC membrane. When LYZ was used as the foulant, its smaller molecular size led to a more severe flux decline. The water fluxes of the PES-TFC and C_4_P_1_-TFC membranes rapidly dropped to 48.3% and 55.7% of their initial values within a short period, respectively. During the early stage of fouling, the water flux of the C_4_P_1_-TFC membrane declined at a relatively high rate, which was associated with its higher initial flux accelerating foulant deposition [58]. However, its more hydrophilic support layer structure with a higher positive charge density still helped mitigate the formation and progression of internal fouling, enabling it to achieve a higher flux recovery ratio of 88.7%. These results indicated that improving the hydrophilicity and reducing the roughness of the support layer could effectively alleviate fouling in the PRO mode, although this strategy still had limitations under severe internal fouling conditions.

The C_4_P_1_-TFC membrane exhibited superior overall antifouling performance compared with the PES-TFC membrane. This indicated that COS/PDA co-deposition modification synergistically enhanced the fouling resistance and cleaning recoverability of the resultant TFC FO membrane by enhancing the hydrophilicity and reducing the surface roughness of both the substrate and the PA layer, while also modulating the surface charge to some extent.

## 4. Conclusions

In this work, a bio-derived COS and mussel-inspired PDA co-deposition strategy was developed to modify PES substrates for the fabrication of high-performance TFC FO membranes. COS is rich in hydroxyl and amino groups, imparting hydrophilicity and antibacterial properties, whereas PDA served as an adhesive platform to stably immobilize COS on the PES substrate through covalent crosslinking. After COS/PDA co-deposition, excessive PDA aggregation was effectively suppressed, substrate roughness and pore blockage were reduced, and a more favorable interfacial environment for subsequent IP was created. MD simulations revealed that COS retarded MPD diffusion through hydrogen bonding and steric interactions. Meanwhile, the hydrophilic co-deposited substrate promoted MPD adsorption at the reaction interface. The coupling of these effects facilitated the formation of thinner, smoother, and more crosslinked PA layers. Consequently, the optimized C_4_P_1_-TFC membrane achieved water fluxes of 42.2 and 23.5 LMH in PRO and FO modes, respectively, which were 43.1% and 40.2% higher than those of the pristine PES-TFC membrane. Its specific salt fluxes decreased to 0.07 and 0.15 g L^−1^, respectively. Moreover, the C_4_P_1_-TFC membrane showed antibacterial rates of 85.7% against *E. coli* and 86.9% against *S. aureus*, together with improved antifouling performance against BSA and LYZ, especially in FO mode. Overall, this work demonstrates that the co-deposition of bio-derived COS and mussel-inspired PDA serves as an effective and facile approach for coupling substrate modification with regulation of the PA layer, thereby enabling the synergistic enhancement of separation, antibacterial, and antifouling properties of FO membranes.

## Figures and Tables

**Table 1:** Intrinsic separation properties of the prepared PES-TFC and C_4_P_1_-TFC membranes compared with other reported PES-based TFC membranes.

Membrane	*A* (LMH Bar^−1^)	*B* (LMH)	*R* (%)	*S* (μm)	Reference
PES-TFC	1.65	0.24	96.36	537	This work
C_4_P_1_-TFC	2.05	0.23	97.32	355	This work
PES-PVP-TFC	0.081	0.012	/	135	[65]
CS-PES-TFC	3.35	3.49	55.07	580	[69]
MAA-PES-TFC	0.031	0.34	/	638	[70]
DAP-PES-TFC	1.7	/	86.5	610	[71]
PES-TFC-M0	0.66	2.26	78.5	1973	[72]
GO/CS-PES-TFC	2.89	13.54	95.1	776	[72]
CNM-PES-TFC	1.79	0.19	91.3	883	[73]
MXenes-PES-TFC	1.21	0.08	/	911	[74]
PES-TFC-1	1.1	0.09	93.2	1096	[75]
SPES/PES-TFC	2.1	0.15	91.3	335	[75]
HA-PES-TFC	1.21	0.55	/	452	[76]

**Table 2:** Detailed comparison of FO performance between the C_4_P_1_-TFC membrane and other PES-based TFC membranes reported in the literature.

Membrane	*J*_w_ (LMH)	*J*_s_ (gMH)	*J*_s_*/J*_w_ (g L^−1^)	Reference
C_4_P_1_-TFC	23.5	3.5	0.15	This work
CS-PES-TFC	19.7	18.0	0.91	[69]
GO/CS-PES-TFC	16.3	2.3	0.14	[72]
CNM-PES-TFC	12.1	3.0	0.24	[73]
MXenes-PES-TFC	15.2	1.62	0.11	[74]
SPES-PES-TFC	18.0	7.2	0.40	[75]
HA-PES-TFC	17.8	6.6	0.37	[76]
PSf/PES-TFC	13.8	12.7	0.92	[77]
SiNPs-PES-TFC	~12.8	~8.5	~0.66	[78]
P-2SO_3_-2NH_2_-PES-TFC	14.7	4.4	0.30	[79]
CPES-PES-TFC	19.1	16.4	0.86	[80]
CAS-PES-TFC	22.7	3.1	0.14	[81]

## Data Availability

Data will be made available on request.

## Supplementary Materials

The following supporting information can be downloaded at: https://www.mdpi.com/article/10.3390/membranes16060186/s1, Section S.1: Optimization of co-deposition parameters for PES substrates; Section S.2: Molecular dynamics simulation; Section S.3: UV detection of the MPD diffusion; Section S.4: Measurement for pure water permeance (PWP) of substrates; Section S.5: FO performance measurement; Section S.6: Measurement for intrinsic separation properties of membranes; Section S.7: Antibacterial performance test of membranes; Section S.8: Antifouling performance test of membranes; Section S.9: Calculation for the degree of crosslinking of the polyamide layer; Figure S1: FO performance of TFC membranes fabricated on substrate modified by different COS and DA co-deposition concentrations: (a) water flux, and (b) reverse salt flux and specific salt flux; Figure S2: FO performance of TFC membranes fabricated on substrate modified by different COS and DA co-deposition time: (a) water flux, and (b) reverse salt flux and specific salt flux; Figure S3: SEM images and AFM images of PES-TFC-SL (a,c) and C4P1-TFC-SL (b,d); (e) WCA of PES-TFC-SL and C4P1-TFC-SL; (f) zeta potential of PES-TFC-SL, C4P1-TFC-SL, PES-TFC-AL, and C4P1-TFC-AL; Table S1: System Composition of MD models.
