# Supplementary material for: Chitooligosaccharide/Polydopamine Co-Deposition Modifying Substrates for High-Performance Forward Osmosis Membranes with Enhanced Antibacterial and Antifouling Properties
Source: Membranes (Basel). 2026 May 28;16(6):186. doi: 10.3390/membranes16060186 (PMC13302806; doi:10.3390/membranes16060186)
Supplement: Supplementary file 1 [file membranes-16-00186-s001.zip › membranes-4312821-supplementary.pdf]

Supplementary Materials

Chitooligosaccharide/Polydopamine Co-Deposition Modifying Substrates for High-Performance Forward Osmosis Membranes with Enhanced Antibacterial and Antifouling Properties

Ming-Xiao Zhang[1], Rui Han[1], Zhen-Liang Xu[1, 2,*], Xin Zhang[1,3], Dibakar Pandaya[1]

[1]*State Key Laboratory of Chemical Engineering, Membrane Science and Engineering R&D Lab, Chemical Engineering Research Center, School of Chemical Engineering, East China University of Science and Technology, 130 Meilong Road, Shanghai 200237, China;*

[2]*Shanghai Electronic Chemicals Innovation Institute, East China University of Science and Technology, Shanghai 200237, China;*

[3]*Beijing Key Laboratory for Membrane Materials and Engineering, Department of Chemical Engineering, Tsinghua University, Beijing, 100084, China.*

*To whom correspondence should be addressed.

Email: chemxuzl@ecust.edu.cn.

1. Detailed information regarding the appendix

S.1 Optimization of co-deposition parameters for PES substrates

(1) Co-deposition concentration

A series of chitooligosaccharide (COS)/polydopamine (PDA)-modified polyethersulfone (PES) substrates were prepared by co-depositing COS and dopamine hydrochloride (DA) with different concentrations at a 1:1 mass concentration ratio for 4 h. Using them as substrates, the corresponding thin-film composite (TFC) forward osmosis (FO) membranes were fabricated via interfacial polymerization (IP) reaction. The water flux, reverse salt flux and specific salt flux of the prepared TFC FO membranes under pressure-retarded osmosis (PRO) and FO modes were measured separately, and the results are presented in Figure S1.

Compared with the PES-TFC membrane, the TFC membranes prepared with a COS/PDA co-deposited coating exhibited higher water flux in both PRO and FO modes. This indicated that the introduction of the COS/PDA coating effectively modified the surface properties of the substrates and optimized the subsequent IP process. With the increase in COS/PDA co-deposition concentration, the membrane water flux first increased and then slightly decreased. The C_1P_1-TFC and C_2P_2-TFC membranes presented relatively higher water flux values. Excessively high concentrations led to the over-deposition of PDA, and the denser and thicker coating increased the permeation resistance of water molecules, resulting in a decline in water flux [1]. The reverse salt flux of modified membranes with different concentrations were generally within the range of 3 to 3.5 g m^{-2} h^{-1} (abbreviated as gMH), which was slightly lower than that of the PES-TFC membrane overall, demonstrating that COS/PDA co-deposition was conducive to maintaining and even improving the salt rejection performance of the membrane. When the co-deposition concentration was excessively high, the reverse salt flux increased significantly, especially in the FO mode, which might be attributed to the excessive inhibition of m-phenylenediamine (MPD) diffusion and the subsequent decrease in the compactness of the polyamide (PA) layer [2]. The specific salt flux showed the same variation trend as the reverse salt flux. Compared with the PES-TFC membrane, the specific salt flux of all TFC membranes prepared on modified substrates was reduced in both FO and PRO modes, indicating that COS/PDA co-deposition modification could effectively enhance the membrane selectivity. The C_1P_1-TFC and C_2P_2-TFC membranes exhibited relatively

lower specific salt flux values. Considering the permeability-selectivity trade-off and material cost, 1 g L^{-1} DA was selected as the basis for subsequent further optimization studies.

(2) Co-deposition time

Using 1 g L^{-1} DA and 4 g L^{-1} COS as the co-deposition system, PES substrates were modified with the co-deposition time set to 1, 2, 4, 6 and 8 h, respectively. Subsequently, the corresponding TFC membranes were fabricated, and their FO performance was characterized, with the results presented in Figure S2.

When the co-deposition time increased from 1 h to 4 h, the water flux increased in both PRO and FO modes. With a further extension to 6 h and 8 h, the water flux subsequently decreased. This phenomenon can be explained by the fact that an insufficient co-deposition time resulted in limited modification of the substrate by COS/PDA, leading to only a slight improvement in surface hydrophilicity. In contrast, an excessively long co-deposition time caused the overgrowth of PDA aggregates, which increased the mass transfer resistance and thus reduced the membrane water permeability. The reverse salt flux decreased slightly with the increase in co-deposition time from 1 h to 6 h, which was attributed to the comprehensive regulation of the adsorption capacity and diffusion rate of amine monomers by the COS/PDA-modified substrates. When the co-deposition time reached 8 h, the reverse salt flux increased significantly. According to previous research findings [3], this was because the excessively large size of PDA aggregates impaired the integrity of the PA layer. The membranes prepared with a 4 h co-deposition time exhibited the lowest specific salt flux in both PRO and FO modes due to their relatively higher water flux and lower reverse salt flux, so 4 h was selected as the optimal co-deposition time for subsequent experiments.

S.2 Molecular dynamics simulation

The molecular dynamics (MD) simulation was performed using the open-source software GROMACS 2020.6 [4], employing the OPLS-AA force field coupled with the TIP3P water model. The compositions of the simulation cell were presented in Table S1, respectively. The initial model was constructed using Packmol. Energy minimization was first performed to eliminate unfavorable contacts. Subsequently, a 10 ns equilibration simulation was conducted in the NPT ensemble to achieve system density equilibration. During the equilibration stage, the pressure was maintained at 0.1 MPa using the Berendsen barostat. This was followed by a 40 ns production simulation in the

NPT ensemble, where the Parrinello–Rahman barostat was utilized to keep the pressure constant at 0.1 MPa. The NoséHoover thermostat was employed to maintain the temperature at 300 K throughout the entire simulation process. The trajectory corresponding to the 20–30 ns period of the production run was used to calculate the mean squared displacement (MSD) and diffusion coefficient (*D*). The short-range neighbor list cutoff, Coulomb cutoff, and van der Waals cutoff were all set to 1.2 nm. Long-range electrostatic interactions were handled via the Particle Mesh Ewald (PME) method, with an interpolation order of 4 and a Fourier grid spacing of 0.16 nm. The simulation results were visualized using the VMD 1.9.3 software [5].

According to the Einstein relation, the diffusion coefficient *D* is proportional to the MSD [6]. The calculation process is illustrated in Equations (S1) and (S2).

$$MSD(\Delta t) = \frac{1}{N_\alpha}\sum_{i=1}^{N_\alpha}\{[r_i(t+\Delta t) - r_i\,(t)]^2\} \tag{S1}$$

$$D = \lim_{\Delta t \to \infty} \frac{MSD(\Delta t)}{2d \cdot \Delta t} \tag{S2}$$

where N_α is the number of molecules, $r_i(t)$ is the coordinate of molecule *i* at time *t*, MSD is the mean squared displacement at a given time *t*, and *d* is the dimensionality of the molecular motion space. For conventional three-dimensional diffusion, $d = 3$.

S.3 UV detection of the MPD diffusion

The absorbance variation of MPD diffused into n-hexane was quantified by the ultraviolet–visible (UV-vis) spectroscopy [7,8]. The substrate was soaked in an appropriate amount of MPD solution for 5 min, after which the excess MPD solution was poured off, and the substrate surface was rolled back and forth with a rubber roller. This procedure was consistent with the fabrication process of TFC membranes. Next, the substrate was immersed in 50 mL of n-hexane. A wavelength of 295 nm was selected as the maximum absorption wavelength. 2 mL of the n-hexane solution was sampled with a pipette every 15 s over 1 min for UV-vis measurements, with the obtained absorbance values used to compare the MPD diffusion rate.

S.4 Measurement for pure water permeance (PWP) of substrates

The pure water permeance (PWP) of the substrates was evaluated using a cross-flow filtration apparatus, with the tests conducted at a pressure of 1 bar and an effective filtration area of 9.1 cm^2. The membranes were pre-conditioned with deionized (DI) water at 1 bar for 30 min to achieve a

stable performance before testing. The test was conducted at a constant temperature of 25 °C, and the PWP (L m^{-2} h^{-1} bar^{-1}, LMH bar^{-1}) was calculated according to Equation (S3):

$$PWP = \frac{\Delta V}{A_n \Delta t \Delta P} \tag{S3}$$

where ΔV (L) means the volume of permeate solution, ΔP (bar) means the transmembrane pressure, A_n (m^2) represents the effective filtration membrane area, and Δt (h) means the test time.

S.5 FO performance measurement

FO membrane performance was evaluated using a laboratory-scale FO system operated at 25 °C with 23.8 cm^2 effective membrane area. DI water was employed as the feed solution (FS), while a 1 mol L^{-1} NaCl solution served as the draw solution (DS). Both solutions were circulated via peristaltic pumps. The salt concentration of the solutions was determined by measuring their electrical conductivity. All tests were conducted in two modes: the PRO mode, where the active layer was oriented toward the draw solution, and the FO mode, with the active layer facing the feed solution. The water flux (J_w, L m^{-2} h^{-1}, LMH) and reverse salt flux (J_s, g m^{-2} h^{-1}, gMH) can be quantified using the following Equations (S4) and (S5), respectively.

$$J_w = \frac{\Delta m}{\rho A_m \Delta t} \tag{S4}$$

$$J_s = \frac{C_t V_t - C_0 V_0}{A_m \Delta t} \tag{S5}$$

where Δm (g) and ρ (g L^{-1}) represent the mass variation and density of FS, A_m (m^2) signifies the effective area of the membrane, Δt (h) corresponds to the test time, C_0 (g L^{-1}) and V_0 (L) represent the initial salt concentration and volume of FS, respectively, C_t (g L^{-1}) and V_t (L) represent the salt concentration and volume of FS over a test time Δt, respectively.

S.6 Measurement for intrinsic separation properties of membranes

A cross-flow filtration unit was utilized to assess membrane intrinsic separation properties. The assessment was conducted at 25 °C and 5 bar. The pure water permeability coefficient (A, L m^{-2} h^{-1} bar^{-1}, LMH bar^{-1}) is calculated based on Equation (S6):

$$A = \frac{\Delta V}{A_n \Delta t \Delta P} \tag{S6}$$

where ΔV (L) means the volume of permeate solution, ΔP (bar) means the transmembrane pressure, A_n (m^2) represents the effective filtration membrane area, and Δt (h) means the collection time.

The salt rejection (R, %) and salt permeability coefficient (B, LMH) were obtained utilizing a feed solution containing NaCl (1 g L^{-1}). These parameters were calculated using Equations (S7) and (S8):

$$R = \left(1 - \frac{C_p}{C_f}\right) \times 100\% \tag{S7}$$

$$\frac{1-R}{R} = \frac{B}{A(\Delta P - \Delta\pi)} \tag{S8}$$

where C_p (g L^{-1}) and C_f (g L^{-1}) are the permeated and feed salt concentration, respectively, $\Delta\pi$ (bar) signifies the osmotic pressure difference.

Given that external concentration polarization (ECP) is generally less significant than ICP in FO membrane systems, the structural parameter (S, μm) is calculated using Equation (S9):

$$S = \frac{D}{J_w}\left(ln\frac{A\pi_{D,b} + B}{A\pi_{F,m} + J_w + B}\right) \times 10^6 \tag{S9}$$

where D (m^2 s^{-1}) means the diffusion coefficient of the NaCl solution at 25 °C, J_w (LMH) denotes the water flux obtained in FO mode, $\pi_{D,b,}$ (bar) and $\pi_{F,m}$ (bar) are the osmotic pressures of DS and FS during the test time, respectively.

S.7 Antibacterial performance test of membranes

Escherichia coli (*E. coli*) and *Staphylococcus aureus* (*S. aureus*) were used as the test strains, and the antibacterial performance of the membranes was evaluated via the spread plate counting method. First, the membrane samples were cut into 1 cm × 1 cm square pieces and sterilized on both sides under ultraviolet light for 60 min. The test bacterial suspensions were diluted to 1×10^6 CFU mL^{-1}, and 2 mL of the diluted bacterial suspension was added to each sterilized sample to be tested, followed by incubation at 37 °C for 12 h. Subsequently, the membrane samples and bacterial suspension were transferred into centrifuge tubes containing 10 mL of sterile phosphate-buffered saline (PBS) and shaken for 30 min to collect the eluate. Then, the eluate was serially ten-fold diluted with sterile PBS solution. A 0.1 mL aliquot of the diluted suspension was evenly spread onto Luria-Bertani (LB) agar medium, which was then incubated at 37 °C for 18 h. The number of colonies was finally counted and recorded via photography. The antibacterial rate (AR, %) of the membranes was calculated using Equation (S10) [9,10]:

$$AR = \left(1 - \frac{N_1}{N_0}\right) \times 100\% \tag{S10}$$

where N_1 and N_0 represent the colony numbers of the experimental group and the control group

(corresponding to the C_4P_1-TFC membrane and PES-TFC membrane in this experiment), respectively.

S.8 Antifouling performance test of membranes

Dynamic fouling tests were conducted using 200 mg L^{-1} bovine serum albumin (BSA, negatively charged) and 200 mg L^{-1} lysozyme (LYZ, positively charged) as model foulants to evaluate the antifouling performance of membranes. The aforementioned FO test unit was adopted, with 1 mol L^{-1} NaCl solution serving as the draw solution, and the tests were conducted in both FO and PRO modes. Firstly, DI water was used as the feed solution for 1 h to reach a steady state, then the initial water flux was recorded. Subsequently, the feed solution was replaced with BSA or LYZ solution, and the dynamic fouling tests were continuously carried out under constant operating conditions. The water flux of the membrane at different time points was recorded in real time at an interval of 10 min for the FO mode or 5 min for the PRO mode, and the ratio of this flux to the initial water flux was defined as the normalized water flux. The dynamic fouling tests were performed for 360 min in the FO mode and 120 min in the PRO mode, respectively. After the fouling tests, the membrane was rinsed with DI water for 30 min. DI water was then reused as the feed solution to measure the membrane water flux. The antifouling performance of the membranes could be evaluated by the flux decline ratio (FDR, %) and flux recovery ratio (FRR, %), which were calculated using Equations (S11) and (S12):

$$FDR = \frac{J_{w,0} - J_{w,e}}{J_{w,0}} \quad \text{(S11)}$$

$$FRR = \frac{J_{w,r}}{J_{w,0}} \times 100\% \quad \text{(S12)}$$

where $J_{w,0}$ (LMH) is the initial water flux of the membrane, $J_{w,e}$ (LMH) is the water flux at the end of the fouling test, and $J_{w,r}$ (LMH) is the recovered water flux of the membrane after cleaning.

S.9 Calculation for the degree of crosslinking of the polyamide layer

The degree of crosslinking (DC, %) of the polyamide layer was calculated by the O/N atomic ratio using Equations (S13) and (S14), respectively.

$$\frac{O}{N} = \frac{3m + 4n}{3m + 2n} = r \quad \text{(S13)}$$

$$DC = \frac{m}{m + n} \times 100\% = \frac{4 - 2r}{1 + r} \times 100\% \quad \text{(S14)}$$

where m and n represent the crosslinked and linear structures of the polyamide layer, respectively [11].

2. Supplementary Tables

Table S1. System composition of MD models.

System	Number of molecules		
	H_2O	MPD	COS
MPD-H_2O	6000	20	0
MPD-COS-H_2O	6000	20	2

3. Supplementary Figures

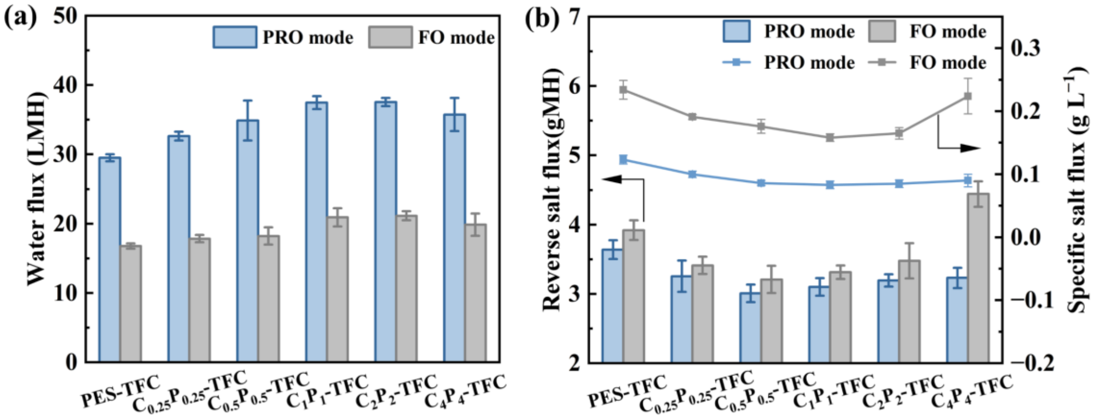

Figure S1. FO performance of TFC membranes fabricated on substrate modified by different COS and DA co-deposition concentrations: (a) water flux, and (b) reverse salt flux and specific salt flux.

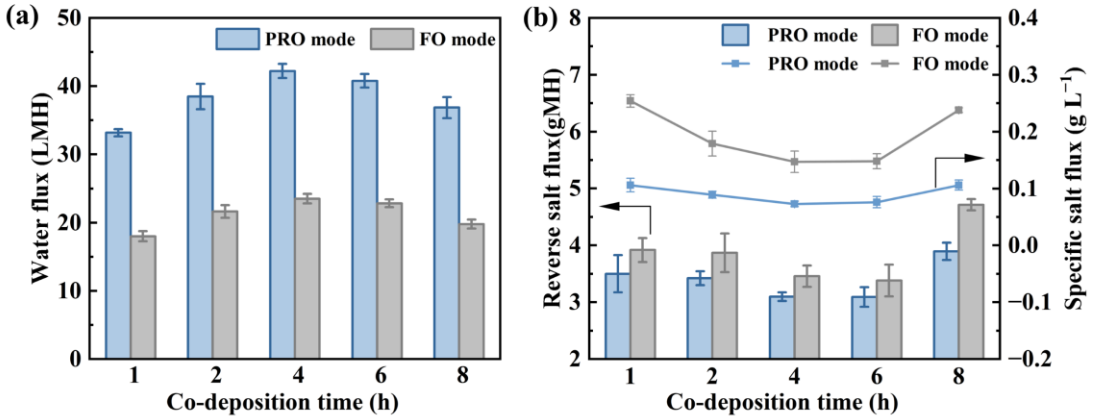

Figure S2. FO performance of TFC membranes fabricated on substrate modified by different COS and DA co-deposition time: (a) water flux, and (b) reverse salt flux and specific salt flux.

To investigate the antifouling performance of the membranes in FO and PRO modes, the hydrophilicity, morphology and roughness of bottom surface of the PES-TFC and C_4P_1-TFC membranes (PES support layer side, denoted as PES-TFC-SL and C_4P_1-TFC -SL) as well as the zeta potentials of both top and bottom surfaces were characterized. This work was based on the known

hydrophilicity, morphology and roughness of the top surfaces of PES-TFC and C_4P_1-TFC membranes (PA active layer side, denoted as PES-TFC-AL and C_4P_1-TFC -AL). The test results are presented in Figure S3.

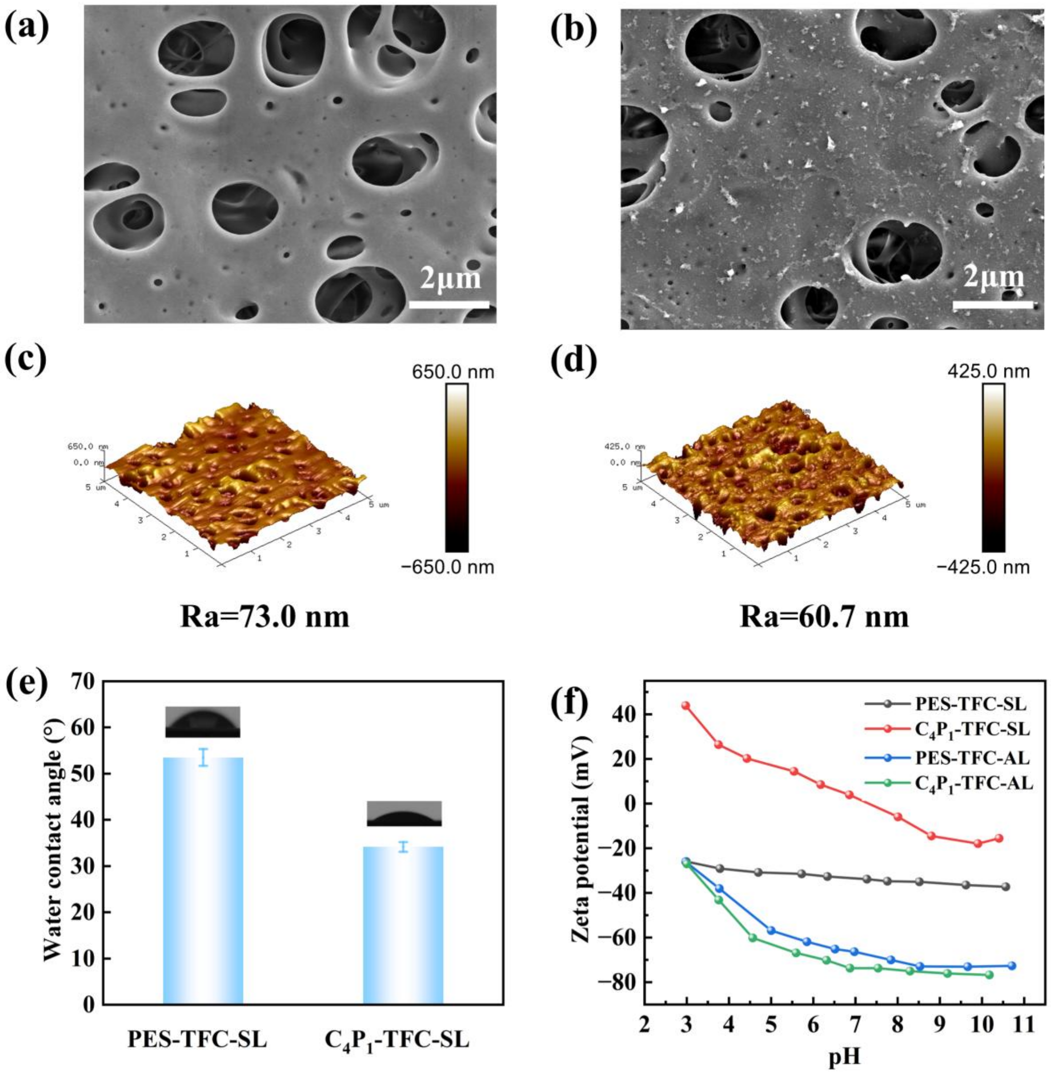

Figure S3. SEM images and AFM images of PES-TFC-SL (a, c) and C_4P_1-TFC-SL (b, d); (e) WCA of PES-TFC-SL and C_4P_1-TFC-SL; (f) zeta potential of PES-TFC-SL, C_4P_1-TFC-SL, PES-TFC-AL, and C_4P_1-TFC-AL.

References

1. Shen, Q.; Lin, Y.; Ueda, T.; Zhang, P.; Jia, Y.; Istirokhatun, T.; Song, Q.; Guan, K.; Yoshioka, T.; Matsuyama, H. The underlying mechanism insights into support polydopamine decoration toward ultrathin polyamide membranes for high-performance reverse osmosis. *J. Membr. Sci.* **2022**, *646*, 120269. doi:https://doi.org/10.1016/j.memsci.2022.120269.

2. Shi, X.; Zhang, Q.; Wang, Z.; Bi, Q.; Lin, Y. Acetone-modulated reverse interfacial polymerization was employed to prepare PEI/PDA positively charged composite nanofiltration membranes for Mg^{2+}/Li+ separation. *J. Membr. Sci.* **2025**, *720*, 123780. doi:https://doi.org/10.1016/j.memsci.2025.123780.
3. Zhu, J.; Tsehaye, M.T.; Wang, J.; Uliana, A.; Tian, M.; Yuan, S.; Li, J.; Zhang, Y.; Volodin, A.; Van der Bruggen, B. A rapid deposition of polydopamine coatings induced by iron (III) chloride/hydrogen peroxide for loose nanofiltration. *J. Colloid Interface Sci.* **2018**, *523*, 86-97. doi:https://doi.org/10.1016/j.jcis.2018.03.072.
4. Abraham, M.J.; Murtola, T.; Schulz, R.; Páll, S.; Smith, J.C.; Hess, B.; Lindahl, E. GROMACS: High performance molecular simulations through multi-level parallelism from laptops to supercomputers. *SoftwareX* **2015**, *1-2*, 19-25. doi:https://doi.org/10.1016/j.softx.2015.06.001.
5. Humphrey, W.; Dalke, A.; Schulten, K. VMD: Visual molecular dynamics. *J. Mol. Graph.* **1996**, *14*, 33-38. doi:https://doi.org/10.1016/0263-7855(96)00018-5.
6. Yan, M.; Xi, Y.; Jiang, N.; Li, Q.; Zheng, S.; Hu, Y.; Liu, Y.; Bao, W.; Huang, M. High-performance thin film composite forward osmosis membrane for efficient rejection of antimony and phenol from wastewater: Characterization, performance, and MD-DFT simulation. *J. Membr. Sci.* **2024**, *703*, 122847. doi:https://doi.org/10.1016/j.memsci.2024.122847.
7. Xiao, F.; Ge, H.; Wang, Y.; Bian, S.; Tong, Y.; Gao, C.; Zhu, G. Novel thin-film composite membrane with polydopamine-modified polyethylene support and tannic acid-Fe^{3+} interlayer for forward osmosis applications. *J. Membr. Sci.* **2022**, *642*, 119976. doi:https://doi.org/10.1016/j.memsci.2021.119976.
8. He, M.; Zhang, W.; Zhang, H.; Lian, J.; Gao, Y.; Wang, J.; Lv, Y.; Wang, X.; Miao, R.; Wang, L. Controlling amine monomers via UiO-66-NH_2 defect sites to enhance forward osmosis membrane performance for lithium recovery. *Chem. Eng. J.* **2024**, *493*, 152321. doi:https://doi.org/10.1016/j.cej.2024.152321.
9. Geng, Z.; Zhao, X.; Fan, Y.; Wang, C.; Huo, H.; Yang, X.; Cai, Y.; Wang, C.; Su, Z. Multi-functional Ag@NH_2-UiO-66/PAES-COOH self-supporting symmetric

hybrid membrane for forward osmosis separation. *J. Membr. Sci.* **2023**, *675*, 121538. doi:https://doi.org/10.1016/j.memsci.2023.121538.

10. Chen, G.-E.; Zhao, S.-N.; Shi, Y.-N.; Xu, Z.-L.; Ye, J. Superhydrophilic and antibacterial PVDF membrane modified by TA-PEI@DMSN nanocomposite for efficient oil-water separation. *J. Environ. Chem. Eng.* **2025**, *13*, 116211. doi:https://doi.org/10.1016/j.jece.2025.116211.
11. Sun, P.-F.; Yang, Z.; Song, X.; Lee, J.H.; Tang, C.Y.; Park, H.-D. Interlayered Forward Osmosis Membranes with $Ti_3C_2T_x$ MXene and Carbon Nanotubes for Enhanced Municipal Wastewater Concentration. *Environ. Sci. Technol.* **2021**, *55*, 13219-13230. doi:https://doi.org/10.1021/acs.est.1c01968.
